# Orthostatic Cognitive Dysfunction in Postural Tachycardia Syndrome After Rapid Water Drinking

**DOI:** 10.3389/fnins.2019.00327

**Published:** 2019-04-09

**Authors:** Belén Rodriguez, Raya Zimmermann, Klemens Gutbrod, Doerthe Heinemann, Werner J. Z’Graggen

**Affiliations:** ^1^Department of Neurosurgery, Inselspital, Bern University Hospital, University of Bern, Bern, Switzerland; ^2^Department of Neurology, Inselspital, Bern University Hospital, University of Bern, Bern, Switzerland

**Keywords:** postural tachycardia syndrome, orthostatic intolerance, cognitive impairment, water intake, alertness, divided attention, working memory

## Abstract

**Background:** Postural tachycardia syndrome (POTS) is a form of autonomic dysregulation and is characterized by an excessive heart rate (HR) increment upon the upright body position while blood pressure is maintained. Patients experience typical symptoms of orthostatic intolerance such as dizziness, nausea and cognitive impairments. The present study assessed position-dependent attentional and cognitive functioning in POTS patients compared to healthy subjects and tested the response of cognitive performance to acute water intake.

**Methods:** Data was obtained from eight patients with neuropathic POTS and eight healthy subjects of similar age and gender. All participants completed questionnaires that assessed health-related quality of life and depression and underwent four rounds of neuropsychological testing overall, each before and after the intake of 500 ml still mineral water and both in the supine and in the upright posture.

**Results:** Postural tachycardia syndrome patients showed deficits in working memory (WM) exclusively in the upright position compared to healthy subjects, but no position-dependent impairments in alertness or divided attention. Rapid water ingestion had a beneficial effect on WM in the upright posture, lead to a decrease in HR increment and to an improvement of subjective symptom experience.

**Conclusion:** The results provide support for the occurrence of purely orthostatic cognitive deficits in POTS, especially when increased executive control and cognitive resources are required and document a favorable effect of water intake on cognitive performance. These findings have important implications for the management of cognitive symptoms in POTS as high water intake is an easy and accessible strategy.

## Introduction

The Postural Tachycardia Syndrome (POTS) is a form of autonomic dysregulation, typically accompanied by symptoms of orthostatic intolerance (OI). OI is a condition characterized by provocation of symptoms upon standing which improve by assuming the supine position (Lambert and Lambert, 2014). POTS is defined in adults by a clinically symptomatic heart rate (HR) increment of 30 beats per minute (bpm) or more within 10 min of standing or head-up tilt (HUT) in the absence of orthostatic hypotension (Grubb, 2008; Freeman et al., 2011). OI symptoms typically include lightheadedness, dizziness, nausea, palpitations, blurred vision, mental clouding and cognitive dysfunction. Patients may also experience non-orthostatic symptoms (e.g., bladder, bowel, and sleep disturbances) (Freeman et al., 2011; Graf et al., 2018). The manifestation of symptoms varies in severity, frequency and pattern, resulting in POTS being a very heterogenous disorder (Deb et al., 2015). The most common subtype of POTS in adults (50%), so called neuropathic POTS, is characterized by a peripheral sympathetic denervation process affecting primarily the lower limbs, typically resulting in excessive venous pooling during standing and loss of sweating in the extremities. In hyperadrenergic POTS patients experience an increased central sympathetic drive with orthostatic plasma norepinephrine levels of at least 600 pg/ml. Other causes include physical deconditioning and volume dysregulation (Benarroch, 2012; Graf et al., 2018). However, the current data on the prevalence of POTS subtypes is not strong.

Cognitive symptoms are reported by 77–96% of POTS patients and experienced by 67% on a daily basis (Deb et al., 2015). Patients typically refer to their cognitive difficulties as “brain fog” or “mental clouding.” It has not yet been clearly defined which cognitive functions are affected, as the descriptions used by patients are often imprecise and previous research resulted in varying neuropsychological profiles. Typically, deficits were found in working memory (WM) (Anderson et al., 2014), cognitive processing speed and executive functions (Ocon et al., 2012), selective and sustained attention (Arnold et al., 2015). The reported cognitive deficits were all limited to the upright position. The exact underlying cause of orthostatic cognitive symptoms still remains elusive. Numerous attempts to link the described impairment to inadequate cerebral perfusion and/or autoregulation showed inconsistent results (Schondorf et al., 2005; Ocon et al., 2009).

Previous studies showed a reduction of HR increment paralleled by subjective improvement of OI symptoms after acute intake of 450–500 ml water within 20–30 min (Z’Graggen et al., 2010) or administration of 1,000 ml IV saline within approximately 60 min (Gordon et al., 2000). However, we are not aware of previous research studying the effect of acute water intake on cognitive symptoms.

The aim of the current study was to evaluate the response of position-dependent cognitive symptoms in POTS to acute water ingestion. We therefore examined alterations of alertness, divided attention (DA) and WM during orthostatic stress in POTS patients and healthy controls with the intervention of rapid water intake.

## Materials and Methods

### Participants

The present study received full local ethical approval (Kantonale Ethikkommission Bern, Switzerland; project-ID: 2017-01368) and was carried out in accordance with the Declaration of Helsinki. The trial was registered at ClinicalTrials.gov (Registration-URL: http://www.clinicaltrials.gov; unique identifier: NCT03253120). Eight patients with confirmed neuropathic POTS (6 female, mean age 25.3, range 18–45 years) and eight healthy control subjects (7 female, mean age 24.4, range 23–28 years) participated. All participants provided full written informed consent. The diagnosis of neuropathic POTS was made according to published criteria (Grubb, 2008; Freeman et al., 2011) and was based upon the patients’ medical history, physical and neurological examination, autonomic function testing, thermoregulatory sweat test and/or quantitative sudomotor axon reflex testing, microneurography, measurement of supine and standing plasma norepinephrine levels and cutaneous biopsy in selected cases. Participants had no dietary restrictions during the days before the study, but had nil by mouth after midnight. All vasoactive and psychotropic medication was stopped at least five half-lives prior to testing.

### Material

#### Questionnaires

To assess the participants’ general health status all subjects completed the following self-report questionnaires: Beck Depression Inventory Second Edition (BDI-II) (Beck et al., 1996), Pittsburgh Sleep Quality Index (PSQI) (Buysse et al., 1989) and 36-Item Short Form Health Survey (SF-36) (Ware and Sherbourne, 1992).

#### Cardiovascular Autonomic Function Testing

All participants underwent standard screening cardiovascular autonomic function testing according to the guidelines of our autonomic unit and according to published guidelines (Ziemssen and Siepmann, 2019). Throughout the experimental procedure beat-to-beat blood pressure (BP) and HR were recorded with the Finapres^®^ NOVA device (Finapres Medical Systems BV, Arnhem, Netherlands) from the non-dominant hand. In addition, a three-lead electrocardiogram was recorded and intermittent brachial BP and HR values were measured using a Dinamap Pro 100 sphygmomanometer (GE Medical Systems, Tampa, FL, United States). HUT testing was performed with a tilt angle of 60° (see below).

#### Neuropsychological Testing

The neuropsychological assessment consisted of the following three subtests of the computer-based and validated test battery “Test of Attentional Performance” (TAP; Zimmermann and Fimm, 2007): (i) “Alertness”: visual reaction time (RT) task with (phasic alertness) and without acoustic warning (tonic alertness), test duration 4 min. (ii) “Divided Attention”: visual and auditory stimuli had to be processed simultaneously, test duration 4.5 min. (iii) “Working Memory”: consisted of a parametric *n*-back test using 2-back levels, test duration 5 min. Subjects responded to each task by pressing a button placed in their dominant hand. For every subtest the required RT and for WM, the total number of false responses and omissions were recorded.

### Study Protocol

**FIGURE 1:**
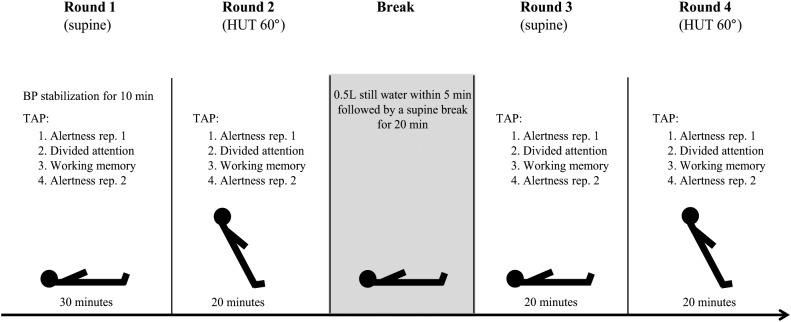
Study protocol. Participants completed four rounds of the same sequence of neuropsychological tests of the test battery “Test of Attentional Performance” (TAP; Zimmermann and Fimm, 2007). BP, blood pressure; rep., repetition; HUT, head-up tilt.

The detailed study protocol is shown in Figure 1. Neuropsychological testing was started after HR and BP remained stable for 10 min in the supine position. Participants were familiarized with each neuropsychological test before completing the first round of tests in the supine position. The same sequence of tests was then repeated in the upright (60° HUT) position. After a short recovery in the supine position, participants were asked to drink 500 ml of commercially available still mineral water (Adelbodner Cristal, Mineralquellen Adelboden AG, Schweiz) at room temperature within 5 min. After 20 min neuropsychological testing was repeated in the supine and upright position. For capturing a possible position- and/or fatigue-dependent decrease in attentional performance over time, participants completed the alertness task at the beginning and at the end of each test round. Participants were asked to rate their subjective experience of selected OI symptoms on a 10-point Likert scale after each HUT.

### Data Analysis

Statistical analyses were performed using SPSS Statistics 25.0 (IBM, Armonk, NY, United States). Group differences in patient characteristics including questionnaires and hemodynamic data were assessed using two-tailed Student’s *t*-test for independent samples and Mann–Whitney *U*-test, respectively. Changes in subjective experience of OI symptoms (sum of OI symptom ratings) during HUT were analyzed using Wilcoxon’s signed-ranks test. For the analysis of neuropsychological data, 2 × 2 × 2 analyses of variance (ANOVA) for repeated measures with *post hoc* Bonferroni correction for multiple comparisons were conducted. The factors were (i) group (POTS patients; healthy control subjects), (ii) body position (supine; HUT), and (iii) water intake (before water intake; after water intake). All statistical analyses of RTs were based on individual median values. False responses and omissions during the WM subtest were processed as absolute values and due to small ranges added up into one score (=error rate). To test for a possible change in performance over time, a paired-samples *t*-test for both repetitions of the alertness subtest in each round of tests was implemented. Group data are reported as mean ± SD. A two-tailed *P*-value ≤ 0.05 was defined as statistically significant. To determine the sample size, an *a priori* power analysis was performed based on previous neuropsychological data in POTS (Ocon et al., 2012; Anderson et al., 2014; Arnold et al., 2015) using G^∗^Power (Erdfelder et al., 1996). The analysis indicated that a total sample of 16 people would be needed to detect large effects (Cohen’s *d* = 0.8) with an 80% power using a mixed model ANOVA for repeated measures with alpha set at 0.05 with two experimental groups and four measurements each.

## Results

### Participant Characteristics

Demographics of patients and healthy control subjects and results of questionnaires are summarized in Table 1. Detailed results of autonomic function testing are shown in Table 2. Cardiovascular autonomic function testing revealed normal results of supine sympathetic and parasympathetic function tests both in POTS patients and controls. In POTS patients water intake resulted in a significantly reduced HR increment (before: 41.50 ± 10.14 bpm; after: 24.88 ± 9.25 bpm, *p* < 0.001), whereas no such difference was noticed in healthy subjects (before: 11.88 ± 4.12 bpm; after: 17.75 ± 6.58 bpm, *p* = 0.152).

**Table 1:** Participant characteristics and questionnaires.

	Control (*n* = 8)	POTS (*n* = 8)	*P*-value
Age *Mean* (*Range*)	24.4 (23-28)	25.3 (18-45)	0.234
Gender *f* (*%*)	7 (88%)	6 (75%)	
BDI-II	7 (8.09)	6.63 (7.61)	0.925
PSQI	3.88 (2.75)	6.75 (2.60)	0.050^∗^
SF-36 physical	57.46 (3.89)	43.41 (7.29)	0.003^∗∗^
SF-36 psychological	47.87 (10.11)	46.42 (10.58)	0.645
Orthostatic symptoms before H_2_O	19.38 (3.50)	50.75 (23.19)	<0.001^∗∗∗^
Orthostatic symptoms after H_2_O	19.00 (3.85)	36.63 (18.22)	0.003^∗∗^


*Values are given as mean (standard deviation) if not differently characterized. POTS, postural tachycardia syndrome; f, female; BDI-II, Beck Depression Inventory II; PSQI, Pittsburgh Sleep Quality Index; SF-36, Short Form (36) Health Survey. ^∗^*P* ≤ 0.05; ^∗∗^*P* ≤ 0.01; and ^∗∗∗^*P* ≤ 0.001*

**Table 2:** Results of cardiovascular autonomic function testing.

	Control (*n* = 8)	POTS (*n* = 8)	*P*-value
HR variability deep breathing	23.59 (6.03)	23.06 (6.61)	0.959
Valsalva ratio	1.87 (0.29)	2.3 (0.66)	0.195
Cold pressor			
ΔSBP	31.88 (6.40)	29.16 (7.28)	0.442
ΔDBP	21.88 (8.48)	23.75 (7.27)	0.798
ΔHR	1.75 (15.32)	3.25 (9.38)	0.878
SBP supine before H_2_O	115.75 (10.36)	121.63 (10.85)	0.287
DBP supine before H_2_O	66.50 (4.41)	70.38 (3.24)	0.650
SBP HUT before H_2_O	117.13 (11.95)	123.25 (11.97)	0.323
DBP HUT before H_2_O	74.25 (7.44)	78.63 (6.09)	0.219
HR supine before H_2_O	68.25 (9.96)	69.50 (10.43)	0.810
HR HUT before H_2_O	80.13 (10.10)	111.00 (9.38)	<0.001^∗∗∗^
Change HUT before H_2_O			
ΔSBP	1.38 (7.96)	1.63 (7.67)	0.950
ΔDBP	7.75 (5.78)	8.25 (3.54)	0.838
ΔHR	11.88 (4.12)	41.50 (10.14)	<0.001^∗∗∗^
SBP supine after H_2_O	115.13 (10.93)	121.88 (12.10)	0.261
DBP supine after H_2_O	68.75 (3.73)	71.63 (5.73)	0.254
SBP HUT after H_2_O	119.38 (9.55)	122.75 (11.62)	0.536
DBP HUT after H_2_O	77.38 (6.09)	80.38 (8.07)	0.415
HR supine after H_2_O	58.38 (5.83)	68.00 (8.79)	0.022^∗^
HR HUT after H_2_O	76.13 (8.85)	92.88 (9.88)	0.003^∗∗^
Change HUT after H_2_O			
ΔSBP	4.25 (7.55)	0.88 (9.22)	0.437
ΔDBP	8.63 (7.69)	8.75 (5.23)	0.970
ΔHR	17.75 (6.58)	24.88 (9.25)	0.098


*Values are given as mean (SD). HR variability was calculated as the mean difference in HR between the end of inspiration and the end of expiration during six respiratory cycles at a frequency of 0.1 Hz (results are given in bpm), “supine” refers to values just before 60° head-up tilt (HUT), “HUT” refers to values after 10 min of HUT and Δ values refer to the difference in corresponding supine and HUT values. POTS, postural tachycardia syndrome; SBP, systolic blood pressure (mmHg); DBP, diastolic blood pressure (mmHg); HR, heart rate (bpm); HUT, head-up tilt. ^∗^*P* ≤ 0.05; ^∗∗^*P* ≤ 0.01; and ^∗∗∗^*P* ≤ 0.001*.

### Neuropsychological Testing

**FIGURE 2:**
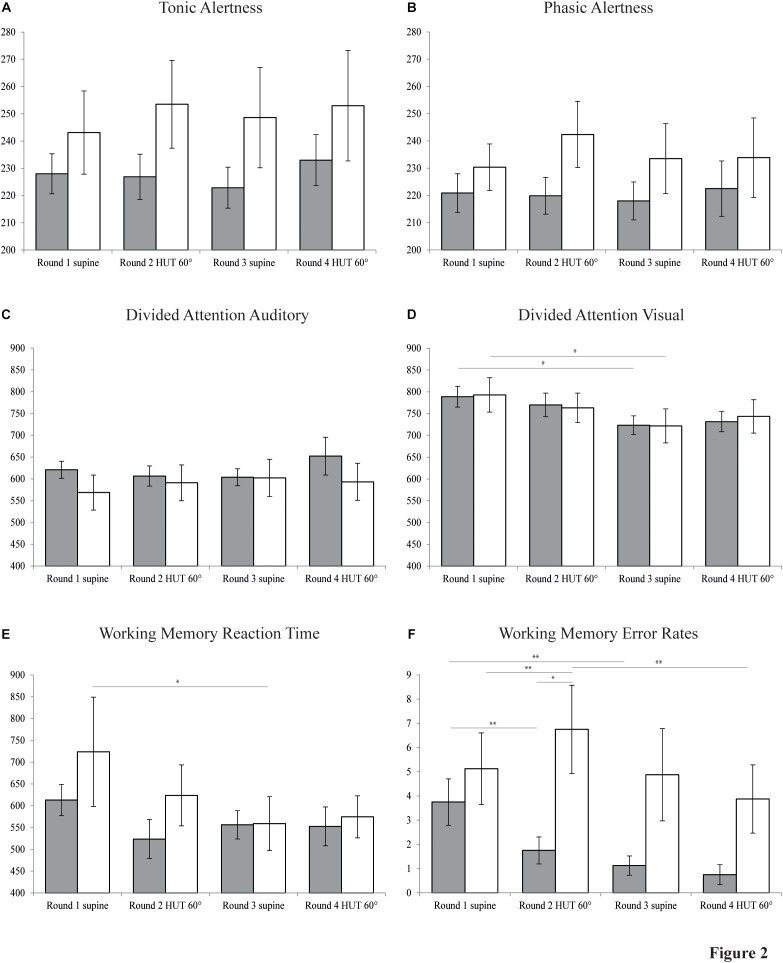
Neuropsychological data. Bar graphs showing results of four rounds of neuropsychological testing in patients with POTS (white bars) and control subjects (gray bars) in the supine and 60° head-up tilt (HUT) position, each before and after the intake of water. **(A)** Tonic alertness. **(B)** Phasic alertness. **(C)** Divided attention auditory. **(D)** Divided attention visual. **(E)** Working memory reaction time. **(F)** Working memory error rates. *Y*-axes of plots A to E indicate reaction time (ms). *Y*-axis of plot F indicates cumulative number of errors and omissions. Values are given as means ± S.E.M. ^∗^*P* ≤ 0.05; ^∗∗^*P* ≤ 0.01.

All neuropsychological data is illustrated in Figure 2. Numerical data is given in Table 3. Phasic alertness showed no significant main effects for group, body position and water intake or interactions between the three factors. The subtest tonic alertness also revealed no significant main effects for either of the factors, but a significant three-way interaction [*F*(1,14) = 6.62, *p* = 0.022]. There was a tendency for slightly longer RTs in POTS patients compared to controls in both tonic and phasic alertness. The paired-samples *t*-tests of the two repetitions of alertness performed at the beginning and the end of each round of neuropsychological tests showed no significant differences of RT neither in phasic nor tonic alertness. Therefore, the second repetitions of the subtest alertness were not included in further analyses.

**Table 3:** Results of neuropsychological testing.

	Control (*n* = 8)	POTS (*n* = 8)
**Alertness tonic** (RT)		
Round 1 (supine)	228 (20.7)	243.13 (43.24)
Round 2 (HUT)	226.88 (23.49)	253.5 (45.56)
Round 3 (supine)	222.88 (21.25)	248.63 (52.1)
Round 4 (HUT)	233 (26.47)	253 (57.33)
**Alertness phasic** (RT)		
Round 1 (supine)	220.87 (19.97)	230.38 (24.04)
Round 2 (HUT)	219.88 (19.07)	242.38 (34.36)
Round 3 (supine)	218 (19.70)	233.5 (36.31)
Round 4 (HUT)	222.5 (28.7)	233.88 (41.21)
**DA auditory** (RT)		
Round 1 (supine)	620.75 (55.65)	568.66 (113.46)
Round 2 (HUT)	606.5 (65.3)	590.87 (116.4)
Round 3 (supine)	603.75 (54.71)	602.13 (120.67)
Round 4 (HUT)	652.16 (122.53)	593.16 (120.2)
**DA visual** (RT)		
Round 1 (supine)	788.75 (66.95)	792.88 (111.51)
Round 2 (HUT)	770.0 (76.34)	763.38 (95.38)
Round 3 (supine)	723.5 (61.1)	721.88 (110.78)
Round 4 (HUT)	731.63 (65.33)	743.75 (108.41)
**WM** (RT)		
Round 1 (supine)	613.25 (101.21)	723.75 (354.22)
Round 2 (HUT)	523.5 (126.87)	623.63 (197.87)
Round 3 (supine)	556.25 (91.66)	558.88 (175.21)
Round 4 (HUT)	552.66 (125.88)	574.75 (136.65)
**WM** (error rates)		
Round 1 (supine)	3.75 (2.71)	5.16 (4.19)
Round 2 (HUT)	1.75 (1.58)	6.75 (5.15)
Round 3 (supine)	1.16 (1.16)	4.88 (5.38)
Round 4 (HUT)	0.75 (1.16)	3.88 (3.98)


*Values are given as mean (standard deviation). RTs are given in ms. POTS, postural tachycardia syndrome; RT, reaction time; HUT, head-up tilt; DA, divided attention; WM, working memory.*

Divided attention was analyzed separately for RTs of trials with auditory and visual stimuli. RT of auditory trials showed no significant main effects, but a significant three-way interaction [*F*(1,14) = 7.64, *p* = 0.015]. For RT of trials with visual stimuli, ANOVA for repeated measures revealed a main effect for water intake [*F*(1,14) = 14.59, *p* = 0.002] which, how further pairwise comparisons showed, originated both in control subjects [*F*(1,14) = 6.36, *p* = 0.024] and in POTS patients [*F*(1,14) = 7.53, *p* = 0.016] in the supine position before vs. after water intake, reflecting a possible learning effect.

Working memory RT showed a significant main effect of water intake [*F*(1,14) = 6.37, *p* = 0.024] and a significant two-way interaction of body position^∗^water intake [*F*(1,14) = 7.00, *p* = 0.019]. Separate follow-up 2 × 2 ANOVAs for body position and group further revealed a significant main effect of body position [*F*(1,14) = 4.92, *p* = 0.044] before water intervention, possibly reflecting a learning effect. No such effect was found any more after the intake of water [*F*(1,14) = 0.12, *p* = 0.740]. WM error rates indicated a main effect of water intake [*F*(1,14) = 15.68, *p* = 0.001], a two-way interaction between body position and group [*F*(1,14) = 7.23, *p* = 0.018] and a three-way interaction between all factors [*F*(1,14) = 7.96, *p* = 0.014]. Furthermore, there was a tendency toward a main effect of group on error rates in WM [*F*(1,14) = 3.97, *p* = 0.066].

## Discussion

The present study investigated orthostatic changes in attentional and cognitive performance in POTS patients compared to healthy controls and additionally tested the effect of rapid water intake on these parameters. POTS patients presented with deficits in WM compared to healthy subjects, which were strictly limited to the upright position and improved after water ingestion.

The results show that neither POTS patients nor control subjects qualify for clinical depression (BDI-II score < 8; Beck et al., 1996) and that there are no group differences regarding the BDI-II score, which contrasts results of previous studies (Anderson et al., 2014; Arnold et al., 2015). Based on the low BDI-II scores of our patient collective, we infer that neuropsychological assessment in the present study was most probably not affected by symptoms of depression. In contrast, the PSQI score of >5 in our POTS cohort is indicative of poor sleep quality (Buysse et al., 1989) and also differed significantly from controls. Therefore, a possible negative effect cognitive performance in POTS patients cannot be ruled out. SF-36 scores indicate impaired quality of life in POTS regarding physical, but not mental health.

Purely orthostatic cognitive deficits in POTS were predominantly observed in the *n*-back task of WM, which largely supports the findings of previous studies (Ocon et al., 2012). Regarding alertness and DA, however, there was no position-dependent impairment evident in patients. Controls, in contrast, showed a tendency for improved performance throughout the four repetitions of WM, which can probably be attributed to a learning effect. Analyses of the two repetitions of alertness at the beginning and at the end of each round showed no significant differences, indicating that there was no change in performance due to fatigue.

Hence, cognitive symptoms in POTS appear to be task- and position-specific, suggesting (i) a selective and domain-specific manifestation of cognitive dysfunction and (ii) a functional and not an absolute deficit. Our results indicate that deficits become apparent as the complexity of the task increases and functions requiring greater executive control and cognitive resources are involved. In line with this, a rising position-dependent deficit in WM performance was found in POTS patients as the *n*-back difficulty increased (Ocon et al., 2012). To confirm this assumption future research focusing on more complex tasks requiring higher cognitive functions is needed. It is not possible to definitively conclude if the reported experience of “mental clouding” or “brain fog” in POTS patients are caused primarily by impairments in WM or if higher cognitive functions are affected in general.

In accordance with previous findings the intake of 500 ml water had a positive effect on hemodynamic changes and subjective experience of OI symptoms in POTS (Z’Graggen et al., 2010). Furthermore, our results show a significant improvement in WM performance during HUT after water drinking. The exact mechanisms underlying the beneficial effect of water on tachycardia and OI symptoms are still unknown. It is widely assumed that the water-induced pressor response is not only caused by an increase in fluid volume but also mediated by increased sympathetic activation at the spinal level (Jordan, 2005; Lu et al., 2012), raising the peripheral sympathetic vasoconstrictor discharge rate and thereby reducing peripheral blood pooling and HR increase (Scott et al., 2001). Hence, water intake seems to improve sympathetic functioning, which in turn reduces the subjectively experienced orthostatic stress and ameliorates OI symptoms. There is a broad literature on the effect of experienced stress on cognitive function, discussing different theories concerning the nature of the relationship and presumed involved mechanisms. It has been shown that acute stress is causing an increase in sympathetic tone and also cortisol secretion and leads to impairments in WM and cognitive flexibility (Buchanan et al., 2006). This effect has been observed not only due to physical but also after acute psychosocial stress (Schoofs et al., 2008). Therefore, the improved WM performance after water intake in POTS may be mediated by the reduction in experienced orthostatic stress.

However, the present findings also have their limitations. As the study was conducted with a small sample size it should be considered as a preliminary study, primarily to evaluate suitable neuropsychological testing for assessing cognitive dysfunction in POTS and to explore the possible beneficial effect of water ingestion on cognitive symptoms. Further, more comprehensive research is needed to confirm the effect of water ingestion on cognitive functioning in POTS. Such studies are encouraged to use a testing protocol with a randomized intervention or ideally an additional sham intervention. The results of the present study help future studies to perform a power analysis in order to optimize the sample size. While the inclusion of a control group in our study and the implementation of eight repetitions of the alertness task allowed to control for major learning effects and signs of fatigue, small confounding effects cannot be ruled out. Furthermore, due to the small sample size it was not feasible to control for other confounding effects such as sleep quality in the analysis of neuropsychological data. Lastly, the neuropsychological tests were always presented in a fixed and consequently non-randomized order and thus, the occurrence of sequence effects cannot be excluded.

The present study provides further support for the occurrence of purely orthostatic cognitive deficits, especially when increased executive control and cognitive resources are required and documents a beneficial effect of water intake on cognitive symptoms in POTS. Most importantly, the results provide valuable implications for the management of cognitive symptoms on a daily basis in patients, as high fluid intake in form of water is an easy and accessible strategy.

## Ethics Statement

The present study received full local ethical approval (Kantonale Ethikkommission Bern, Switzerland; project-ID: 2017-01368) and was carried out in accordance with the Declaration of Helsinki. The trial was registered at ClinicalTrials.gov (Registration-URL: http://www.clinicaltrials.gov; unique identifier: NCT03253120). All participants provided full written informed consent.

## Author Contributions

WZ’G, KG, DH, and BR contributed to conception and design of the study. BR, RZ, and WZ’G conducted all experiments and testing. BR performed the statistical analysis. WZ’G, KG, DH, and BR interpreted the results. BR wrote the first draft of the manuscript. WZ’G wrote sections of the manuscript. All authors contributed to manuscript revision, read, and approved the submitted version and provided the approval for publication of the content and agree to be accountable for all aspects of the work in ensuring that questions related to the accuracy or integrity of any part of the work are appropriately investigated and resolved.

## Conflict of Interest Statement

The authors declare that the research was conducted in the absence of any commercial or financial relationships that could be construed as a potential conflict of interest.

## References

[B1] Anderson J. W. Lambert E. A. Sari C. I. Dawood T. Esler M. D. Vaddadi G. (2014). Cognitive function, health-related quality of life, and symptoms of depression and anxiety sensitivity are impaired in patients with the postural orthostatic tachycardia syndrome (POTS). *Front. Physiol.* 5:230. 10.3389/fphys.2014.00230 25009504 PMC4070177

[B2] Arnold A. C. Haman K. Garland E. M. Raj V. Dupont W. D. Biaggioni I. (2015). Cognitive dysfunction in postural tachycardia syndrome. *Clin. Sci.* 128 39–45. 10.1042/cs20140251 25001527 PMC4161607

[B3] Beck A. T. Steer R. A. Brown G. K. (1996). *Beck Depression Inventory-II.* San Antonio, TX: Psychological Corporation.

[B4] Benarroch E. E. (2012). Postural tachycardia syndrome: a heterogeneous and multifactorial disorder. *Mayo Clin. Proc.* 87 1214–1225. 10.1016/j.mayocp.2012.08.013 23122672 PMC3547546

[B5] Buchanan T. W. Tranel D. Adolphs R. (2006). Impaired memory retrieval correlates with individual differences in cortisol response but not autonomic response. *Learn. Mem.* 13 382–387. 10.1101/lm.206306 16741288 PMC1475821

[B6] Buysse D. J. Reynolds C. F. Monk T. H. Berman S. R. Kupfer D. J. (1989). The pittsburgh sleep quality index - a new instrument for psychiatric practice and research. *Psychiatry Res.* 28 193–213. 10.1016/0165-1781(89)90047-4 2748771

[B7] Deb A. Morgenshtern K. Culbertson C. J. Wang L. B. Hohler A. D. (2015). A survey-based analysis of symptoms in patients with postural orthostatic tachycardia syndrome. *Proc (Bayl. Univ. Med. Cent.)* 28 157–159. 10.1080/08998280.2015.11929217 25829642 PMC4365108

[B8] Erdfelder E. Faul F. Buchner A. (1996). GPOWER: a general power analysis program. *Behav. Res. Methods Instrum. Comput.* 28 1–11. 10.3758/BF03203630

[B9] Freeman R. Wieling W. Axelrod F. B. Benditt D. G. Benarroch E. Biaggioni I. (2011). Consensus statement on the definition of orthostatic hypotension, neurally mediated syncope and the postural tachycardia syndrome. *Clin. Auton. Res.* 21 69–72. 10.1007/s10286-011-0119-5 21431947

[B10] Gordon V. M. Opfer-Gehrking T. L. Novak V. Low P. A. (2000). Hemodynamic and symptomatic effects of acute interventions on tilt in patients with postural tachycardia syndrome. *Clin. Auton. Res.* 10 29–33. 10.1007/bf02291387 10750641

[B11] Graf N. Fernandes Santos A. M. Ulrich C. T. Fung C. Raabe A. Beck J. (2018). Clinical symptoms and results of autonomic function testing overlap in spontaneous intracranial hypotension and postural tachycardia syndrome: a retrospective study. *Cephalalgia Rep.* 1:2515816318773774 10.1177/2515816318773774

[B12] Grubb B. P. (2008). Postural tachycardia syndrome. *Circulation* 117 2814–2817. 10.1161/circulationaha.107.761643 18506020

[B13] Jordan J. (2005). Effect of water drinking on sympathetic nervous activity and blood pressure. *Curr. Hypertens. Rep.* 7 17–20. 10.1007/s11906-005-0050-z 15683582

[B14] Lambert E. Lambert G. W. (2014). Sympathetic dysfunction in vasovagal syncope and the postural orthostatic tachycardia syndrome. *Front. Physiol.* 5:280 10.3389/fphys.2014.00280 PMC4112787 25120493

[B15] Lu C. C. Li M. H. Lin T. C. Chen T. L. Chen R. M. Tung C. (2012). Water ingestion reduces skin blood flow through sympathetic vasoconstriction. *Clin. Auton. Res.* 22 63–69. 10.1007/s10286-011-0142-6 22057730

[B16] Ocon A. J. Medow M. S. Taneja I. Clarke D. Stewart J. M. (2009). Decreased upright cerebral blood flow and cerebral autoregulation in normocapnic postural tachycardia syndrome. *Am. J. Physiol. Heart Circ. Physiol.* 297 H664–H673. 10.1152/ajpheart.00138.2009 19502561 PMC2724195

[B17] Ocon A. J. Messer Z. R. Medow M. S. Stewart J. M. (2012). Increasing orthostatic stress impairs neurocognitive functioning in chronic fatigue syndrome with postural tachycardia syndrome. *Clin. Sci.* 122 227–238. 10.1042/cs20110241 21919887 PMC3368269

[B18] Schondorf R. Benoit J. Stein R. (2005). Cerebral autoregulation is preserved in postural tachycardia syndrome. *J. Appl. Physiol.* 99 828–835. 10.1152/japplphysiol.00225.2005 15860686

[B19] Schoofs D. Preuss D. Wolf O. T. (2008). Psychosocial stress induces working memory impairments in an n-back paradigm. *Psychoneuroendocrinology* 33 643–653. 10.1016/j.psyneuen.2008.02.004 18359168

[B20] Scott E. M. Greenwood J. P. Gilbey S. G. Stoker J. B. Mary D. A. (2001). Water ingestion increases sympathetic vasoconstrictor discharge in normal human subjects. *Clin Sci* 100 335–342. 10.1042/cs20000177 11222121

[B21] Ware J. E. Sherbourne C. D. (1992). The mos 36-item short-form health survey (SF-36).1. Conceptual-framework and item selection. *Med. Care* 30 473–483. 10.1097/00005650-199206000-00002 1593914

[B22] Z’Graggen W. J. Hess C. W. Humm A. M. (2010). Acute fluid ingestion in the treatment of orthostatic intolerance - important implications for daily practice. *Eur. J. Neurol.* 17 1370–1376. 10.1111/j.1468-1331.2010.03030.x 20412295

[B23] Ziemssen T. Siepmann T. (2019). The Investigation of the cardiovascular and sudomotor autonomic nervous system—a review. *Front. Neurol.* 10:53 10.3389/fneur.2019.00053 PMC6380109 30809183

[B24] Zimmermann P. Fimm B. (2007). *TAP Testbatterie zur Aufmerksamkeitsprüfung (Version 2.3).* Herzogenrath: Psytest.

